# Effect of bending on radial distribution density, MFA and MOE of bent bamboo

**DOI:** 10.1038/s41598-022-12675-7

**Published:** 2022-05-21

**Authors:** Xuehua Wang, Jingwen Ma, Wei Xu, Benhua Fei, Caiping Lian, Fengbo Sun

**Affiliations:** 1 College of Furnishings and Industrial Design, Nanjing Forestry University, Nanjing, China; 2 International Centre for Bamboo and Rattan, Beijing, China; 3 Jiangsu Co-Innovation Center of Efficient Processing and Utilization of Forest Resources, Nanjing, China

**Keywords:** Materials science, Physics

## Abstract

One of the excellent characteristics of bamboo is the deformation stability. However, the reasons for the good bending stability of bamboo have not been well studied. In this study, we examined the pathways that controls bending deformation in bamboo. A hand-bent *phyllostachys iridescens* member was chosen to examine continuous density distribution, microfibril angle (MFA) and modulus of elasticity (MOE) along radial direction using SilviScan analysis. Our results show that in bent bamboo, MFA is lower in tension sample and higher in compression sample than neutral sample. There is a strong linear positive correlation between density and MOE, while negative linear correlation between MOE and MFA and no obvious linear correlation between MFA and density. Increased bending was influential in primarily changing the MOE, while also altering the density distribution and MFA. Our results demonstrate variation in density, MOE and MFA distribution along radial direction of tension, neutral and compression samples, which play an important role in maintaining the bending characteristics of bamboo.

## Introduction

Bending members are widely used in structures, such as buildings, bridges, furniture, and other fields. Bending lumbers are usually manufactured from straight lumber, which have the ability to spring back as original state changed. However, bending defects, such as wrinkling^1,2^, cross-section deformation^3–5^, and variations in wall thickness^6^ can have negative impact on the bending process^7^ and the bending components safety and service life. As a result, testing, evaluation and analysis of bending defects play an important role in bent member’s application.

Although there has been a great deal of research on bending defect elimination^8^, shape bending accuracy^9^, and crystal structure in bending area^10^ particularly for metals, little research has been reported on bending characteristics of bamboo at present. This is primarily because the practical application of bamboo is very limited. Recently, more research is being carried out to examine the potential utilization of bamboo because of increasing environmental concerns associated steel production. Bamboo has gradually been used in modern architecture^11^, such as Roc Von restaurant in Hanoi, Vietnam, and for other daily necessities^12^, such as bamboo cups. Bending members can make full use of bamboo’s excellent bending property, however; research on bamboo bending characteristics is very limited to support the mechanization and industrialization of bamboo as a bending material. Spring back characteristics in biomass material was considered to be more universal than metal and/or plastic because cavity and hydrophilic hydroxyl groups can promote water absorption and expansion ability^13^. While bamboo also shows good bending shape and stability in practice, little research has been carried out to identify its full potential to replace or complement biomass materials. Due to hollow structures, bamboo tubes have better bending shape that allows more stability than solid in steel. However, hollow structure is not the only attribute that affect stability and there are large differences in other characteristics between bamboo and steel. Bamboo has a hierarchical and anisotropic structure, which showed different characteristics across different directions^14,15^. In addition, Bamboo develops uneven characteristics as density distribution changes radially from outer skin to inner skin^16^, which further complicates the use of bamboo as bending material compared to metals.

Bamboo should be a desirable choice for making bending member if industrialized processing and large–scale commercial applications are promoted. Considering the fast growth and low energy consumption, bamboo can likely replace metals and other biomass products as bending member for large scale commercial application. In this study, we used bamboo, which was bent by hand-made fire-heating method to examine the springback behavior of bamboo under different humidity conditions. We also evaluated spring back behavior of bent bamboo by examining changes in radial density distribution, and the microfibril angle (MFA) and modulus of elasticity (MOE) of inner, middle and outer side of the cross section. Our main objective is to examine how bending affect bamboo and provide important guidelines for realizing bamboo industrialization.

## Materials and methods

### Materials

In this study, we used *Phyllostachys iridescens* obtained from Suoshi Bamboo Inc. The plant *phyllostachys iridescens* used in this research is a very common commercial bamboo species, the collection of this plant comply with relevant institutional, national, and international guidelines and legislation, including IUCN Policy Statement on Research Involving Species at Risk of Extinction and the Convention on the Trade in Endangered Species of Wild Fauna and Flora.

**Figure 1:**
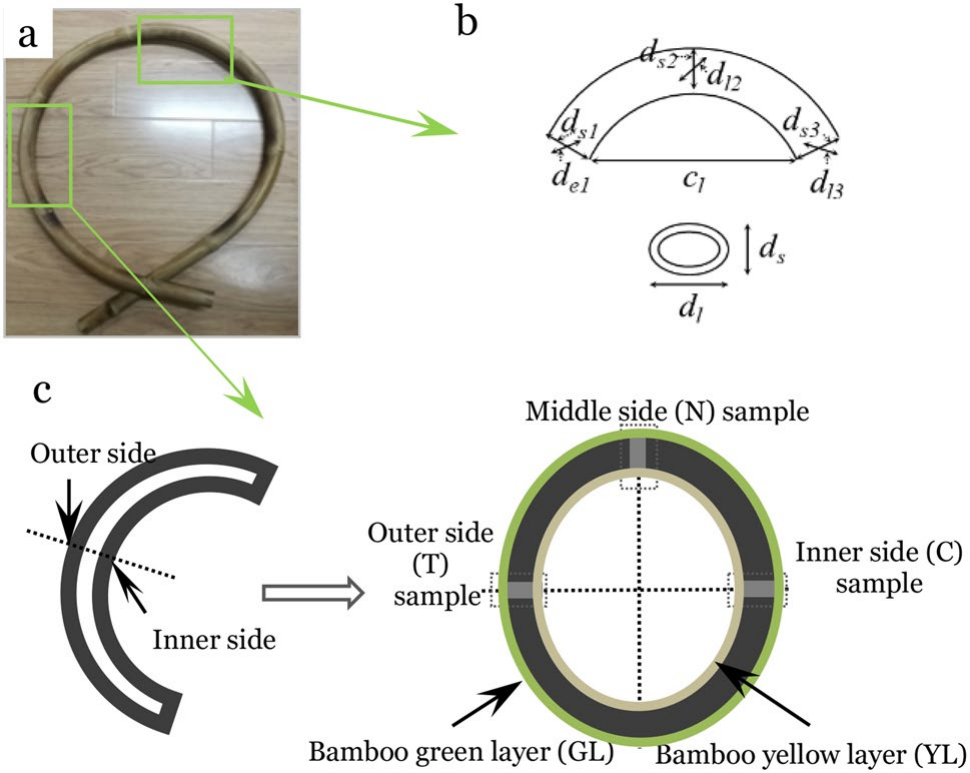
Sampling: (**a**) Curved bamboo; (**b**) sample and dimension measurement for springback behavior: *c*_*l*_ chord length, *d*_*l1*_*, d*_*l2*_*, d*_*l3*_ long diameter on left, middle and right edge; and (**c**) sampling for SilviScan analysis.

The bamboo was 3 ages with culm wall thickness about 4 mm, from Guangde county, Anhui province, China. The *phyllostachys iridescens* was processed with curvature radius around 270 mm (Fig. 1a) by heating over liquid gas flame after holding in hand.

### Bent bamboo springback behavior

**Figure 2:**
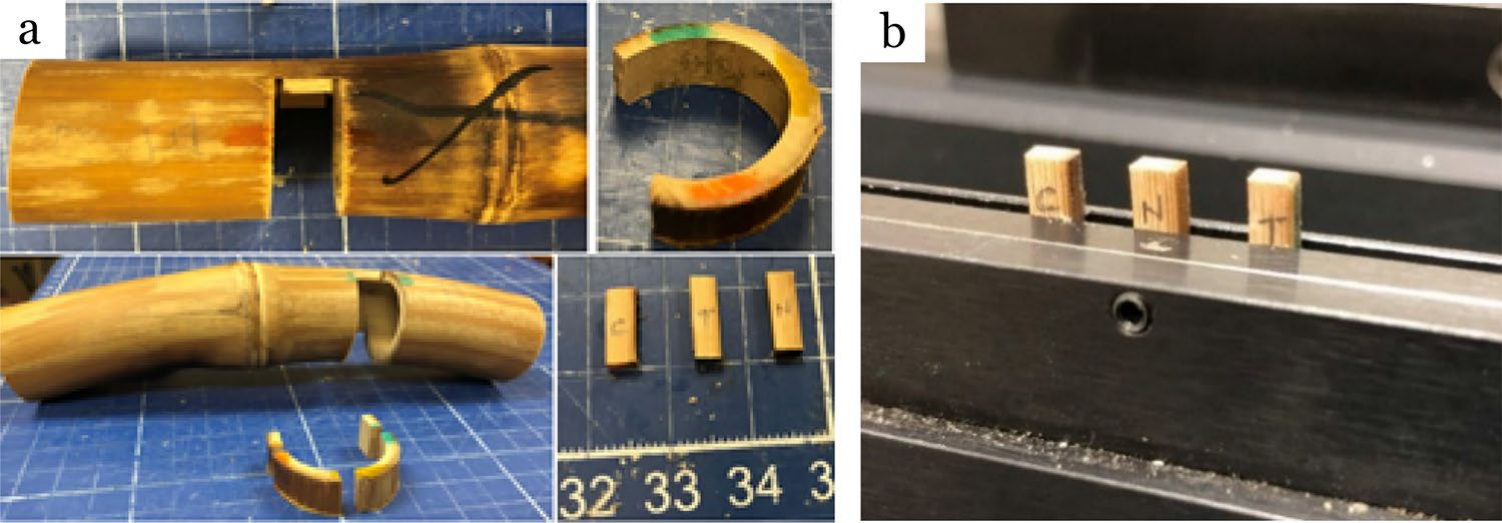
Sample preparation and testing: (**a**) sampling steps from bending bamboo section, (**b**) holding of samples during testing.

Bent bamboo was put in sealed desiccators under three different relative humidity conditions using the saturated salt solutions (Table 1). Desiccators were kept in the environmental chamber at 25 °C to maintain steady relative humidity. Dimension of bent bamboo (Fig. 2) was measured every 24 h for 15 times, then every 96 h for 5 times, and finally every 192 h for 2 times that lasted for a total of 47 days. This was done making sure that the dimension did not change more than 0.5% for the last two times.

**Table 1:** Saturated salt solution and relative humidity conditions.

Saturated salt solution	Relative humidity conditions/%
Magnesium chloride (MgCl_2_)	33
Sodium bromide (NaBr)	59
Potassium sulfate (K_2_SO_4_)	98

The dimension defined as in Fig. 1b was measured. Springback behavior of bent bamboo was calculated as follows:

1 $$R{d}_{l/s}=\frac{\overline{(\frac{{d}_{ln}}{{d}_{sn}})}}{\overline{(\frac{{d}_{l0}}{{d}_{s0}})}}\times 100\%$$

2 $$R{c}_{l}=\frac{{c}_{ln}}{{c}_{l0}}\times 100\%$$

where $R{c}_{l}$*,* $R{d}_{l/s}$ are the ratio of change in chord length and the ratio of long to short diameter with time; respectively. The subscript $n$ stands for the measurement number after the bent bamboo was kept in the desiccator with saturated salt solution, while $0$ is the original value.

### Sample preparation for density, MOR and MOE scanning

Three samples from inner, middle and outer side of bent bamboo were prepared for testing. Based on the stress balance state in bamboo ring, the inner, middle and outer side of bent bamboo samples experienced compression, neutral and tension stress, respectively (Figs. 1c, 2a). The three locations were color marked, and a section of 12 mm longitudinally and about 4/5 circumference was extracted from the culm (Fig. 2a). At each color marked location, a 6 mm (tangentially) piece was sliced off with a sharp thin blade. The samples included the compression side (C), neutral side (N), and tension side (T). The pieces were then cut into strips of 2 mm (tangentially) × 7 mm (longitudinally) × actual thickness (radially) using twin-blade saws.

### X-ray densitometry procedure for density testing

SilviScan analysis was used to measure the radial density distribution. Densitometry was performed on the longitudinal surface of the strips to provide density at a resolution of 25 μm. Bamboo density for each strip was scanned using the X-ray densitometry. Densitometric measurement follows Beer’s Law, which states that the intensity of an x-ray beam that passes through a sample falls off exponentially with sample thickness, and the extent of attenuation is related to the density of the sample (Eq. 3):

3 $$I={I}_{0}{e}^{{-\mathrm{\alpha }}_{m}DT}$$

where ${I}_{0}$ and $I$ are the intensity of incident and transmitted x-ray beam; respectively, $D$ and $T$ are the density and thickness (i.e., the distance that x-ray travels) of sample; respectively, and ${\mathrm{\alpha }}_{m}$ is the mass absorption coefficient.

### X-ray diffractometry procedure for MFA testing

SilviScan analysis was used to measure the radial MFA distribution. Diffractometry was performed on the longitudinal surface at 0.1 mm resolution. Each strip was scanned for MFA using X-ray diffractometry. The MFA is estimated using the relationship between the variance of (cellulose I (002) azimuthal diffraction profile and the microfibril orientation distribution. The (002) diffraction patterns are obtained from the planes whose normal is perpendicular to the microfibril axis. It has shown that the variance (${S}^{2}$) of the (002) azimuthal diffraction profile is related to the MFA ($\mu$) and the variance (${\sigma }^{2}$) of the microfibril orientation distribution as given below^17^:

4 $${S}^{2}\approx \frac{{\mu }^{2}}{2}+{\sigma }^{2}$$

The total variance of the profile (Eq. 4) is estimated as a function of the average MFA and the dispersion of microfibril orientation. MFA was acquired in integral mode, where MFA is averaged within segments along the sample, with a 0.1 mm segment.

### Combined analysis for MOE

Density (*D*) from X-ray densitometry and the coefficient of variation of the intensity of the X-ray diffraction profile (*I*_*CV*_) are combined to compute the fibre MOE^18^:

5 $$MOE=A{({I}_{CV}D)}^{B}$$

The ${I}_{CV}$ is the scattering from cell wall constituents. The model contains two statistically determined calibration constants ($A$ and $B$), that are insensitive to species, and relate to the sonic resonance method used for calibration^18^. This implies that the calculated MOE SilviScan represent a dynamics state.

## Results and discussion

### Bent bamboo springback behavior

**Figure 3:**
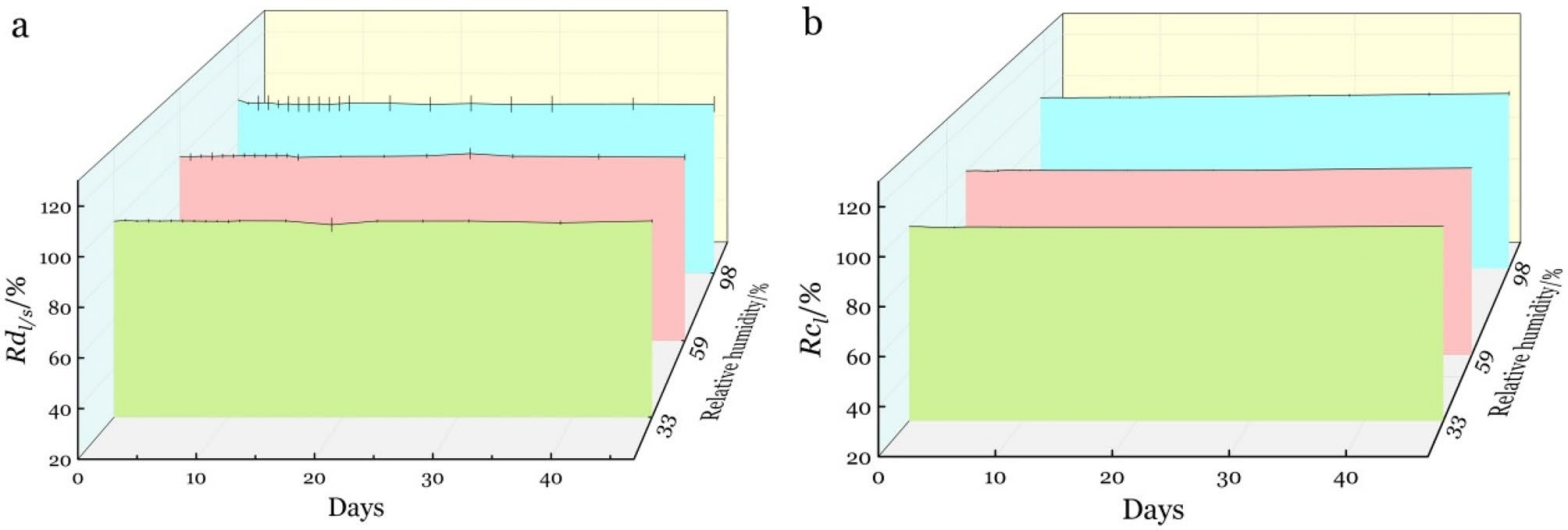
Changes in ratio with time for different relative humidity: (**a**) ratio of long diameter to short diameter (*Rd*_*l/s*_), and (**b**) chord length (*Rc*_*l*_).

Changing of *Rd*_*l/s*_ and *Rc*_*l*_ under different relative humidity with time is shown in Fig. 3. Chord length and ratio of long diameter to short diameter changed within 2% after 47 days under 33% RH, 59% RH, 98% RH, which indicate that bent bamboo springs back little and has a high dimensional stability under low or high humidity conditions.

Containing large amounts of silicon compounds^19^, bamboo has a smooth and high hydrophobicity surface^20^ which resulted in maintaining the bamboo bent stability. We found limited dimensional changes of *Rd*_*l/s*_, *Rc*_*l*_ with time because of viscose deformation recovery under high humidity in the biomaterial bamboo^21^.

### Radial distribution of density, MFA and MOE

**Figure 4:**
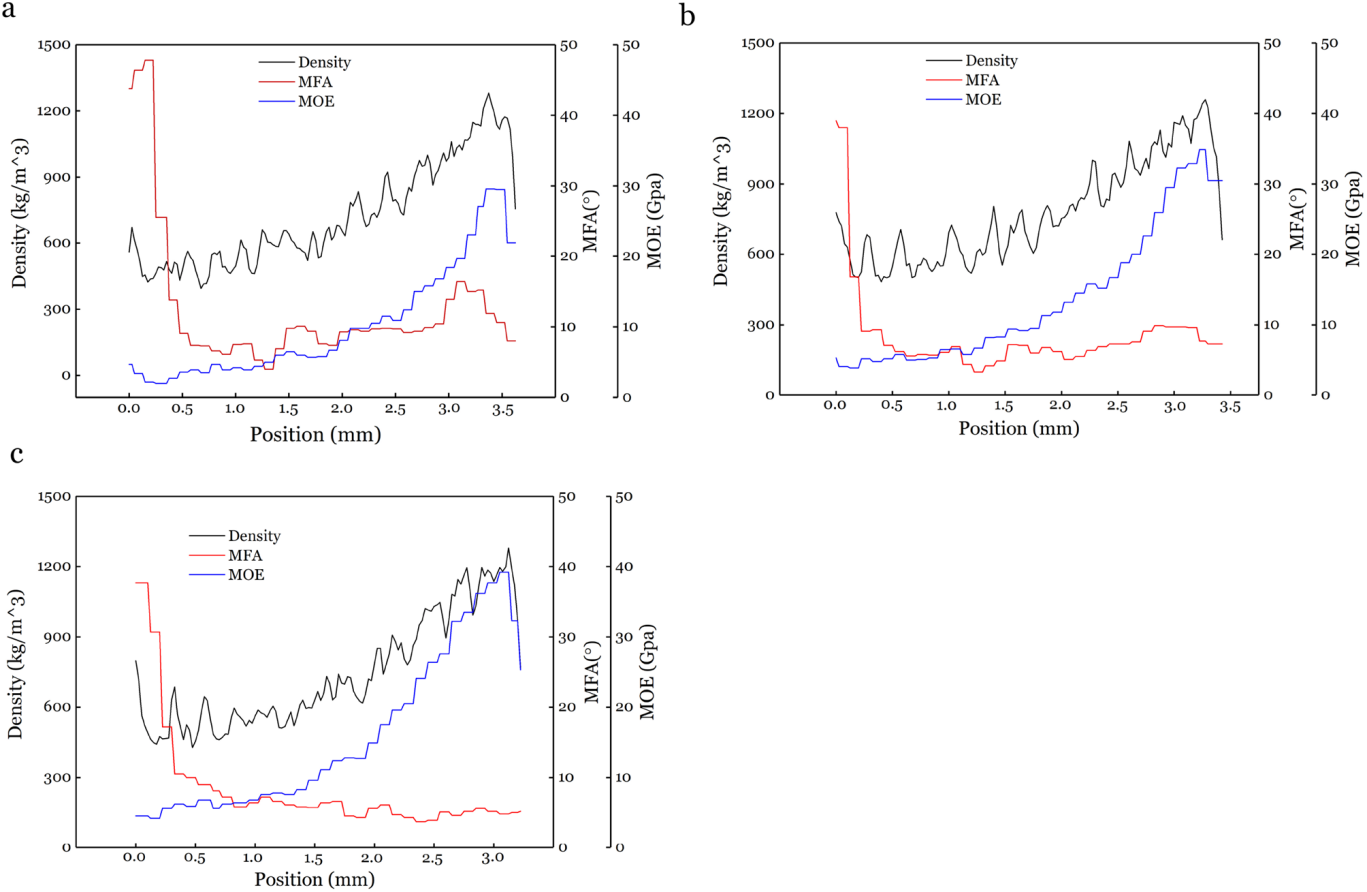
Radial distribution of density, MFA and MOE from YL to GL on (**a**) C sample, (**b**) N sample, and (**c**) T sample.

Radial distribution of density, MFA and MOE on C, N and T of bending bamboo section are shown in Fig. 4. Results show that the distribution along radial of density, MOE and MFA is similar, which MOE increased from bamboo yellow layer (YL) to bamboo green layer (GL), while MFA is much higher in YL compared to GL for all three samples.

### Density distribution along radial direction

**Figure 5:**
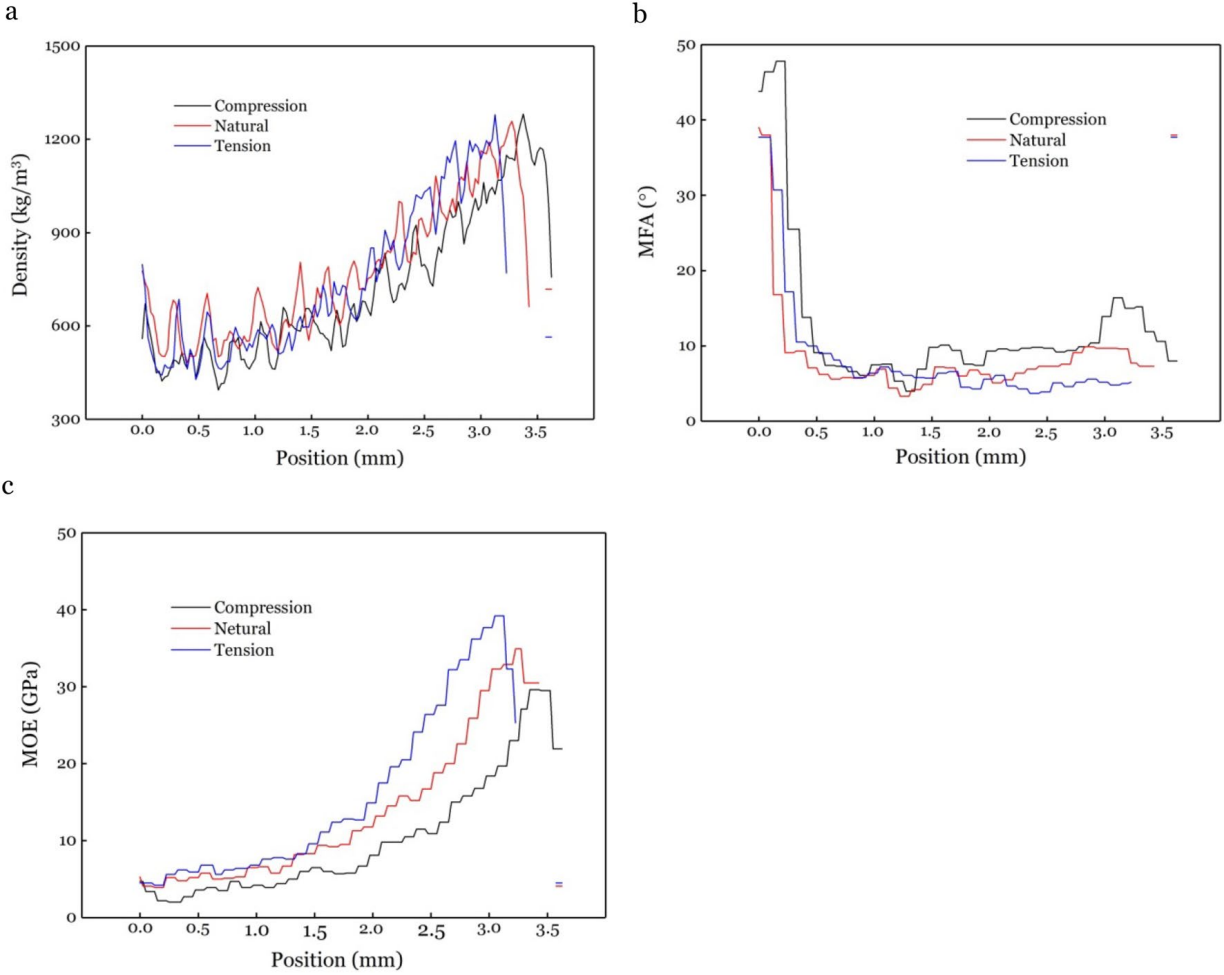
Density (**a**), MFA (**b**) and MOE (**c**) distribution along radial direction from bamboo yellow to bamboo green of different bamboo cross-section parts.

Density distributions along radial direction from YL to GL for C, N and T sample in bending bamboo are showed in Fig. 5a. A similar tendency of increasing density from YL to GL can be found in all three samples, with the density distribution of the C sample lower than the N sample. Arithmetic mean density values for C, N and T samples are calculated as 720.38 kg/m^3^, 775.97 kg/m^3^ and 742.75 kg/m^3^ separately, indicating that the mean density of C and T samples decreased compared with N sample in bending bamboo. The mean density of compression and tension wood also differed from tension wood, which showed positive correlation between density and tension wood fibres percentage^22,23^. Density generally correlate strongly with wood shrinkage^24^. The decrease of density in compression and tension part was helpful in improving the dimensional stability of bent bamboo.

Strain would come with increasing stress when material experience any kind of external force. In some cases, stress is not large enough to produce obvious deformation, but it is difficult to precisely measure stress and strain inside wood, bamboo, or other materials. In general, when there is large stress, strain is visible and can be measured more accurately. Bending is a kind of obvious strain with the material experiencing bending load ultimately resulting in bending section deformation^25,26^. Tube section would change from circular to elliptical after bending^27^, which is a function of different degrees and forms of pore deformation. Following pore deformation, section density redistribute (Fig. 5a), which result in stress redistribution on the bending section of inner, middle and outer side^28^.

### MFA distribution along radial direction

MFA is defined as an inclination of microfibrils from the longitudinal axis, which play an important role in determining the final mechanical properties of bamboo^29^ and wood^30^. There are a lot of factors which may affect MFA in wood or bamboo, such as ages^29,31^, species^32^, and locations in cross-Sect. ^31^. Although previous research has been carried out on MFA distribution along radial direction by picking some points on the cross section^31^, little research has been done to examine the continuous distribution of MFA along radial direction.

MFA distributions along radial direction at a 0.1 mm resolution for sample C, N and T are shown in Fig. 5b. The MFA were highest (38° ~ 48°) in about 0.2 ~ 0.5 mm near YL, followed by sharp decrease (10° ~ 4°) in distance of 0.5 ~ 1.8 mm from YL. A small increase was saw in the last 1 mm for C and N, except T. Arithmetic mean MFA values for C, N and T are calculated as 12.5°, 8.3° and 8.3° in the whole bamboo wall thickness. Although MFA showed nearly same arithmetic mean value in samples N and T, the value of the outer half bamboo wall near the green part experienced the greatest bearing strength^32^. The C, N and T near the GL were calculated as 11.0°, 7.5° and 5.0° with larger distance between the three MFA distribution.

MFA plays an important role in physical properties, such as bending strength, Young’s modulus^29^, and is also an effective character to show bamboo internal stress changing. Previous studies have shown a decrease in MFA with an increase in tension stress^32^, and an increase in MFA with an increase in compression^33^. This agrees with the result of this study that shows these changes mainly occurred in the half outer bamboo wall. A decrease in MFA is represented by better alignment of cellulose along axis direction, which resulted in higher tensile strength^34^. Likewise, an increase in MFA is represented by an increase longitudinal compression, which resulted in better bendability^35^. This changing tendency of the MFA in the outer half T and C wall indicate that bamboo has good bending stability.

### MOE distribution along radial direction

Materials suffer interior stress when bending is carried out with external loading force. However, when the external force is removed, there would be a springback tendency for the bending material caused by the interior stress. This bending springback property can be altered by curvature radius^36,37^, bending method^37^, and the mechanical properties of the bending material^38,39^. As one of the most important mechanical properties, MOE has a negative correlation with bending springback property for bending members^40,41^.

Uneven MOE distribution for C, N and T parts along radial direction is shown in Fig. 5c. The MOE rises with increasing distance from YL to GL for these three samples. The changes in MOE is relatively small at first, followed by large changes in the middle and ultimately dropping again in the last 0.2 mm. MOE value differs in order of T > N > C, which arithmetic mean value calculated as 15.72 GPa, 13.58 GPa, and 10.14 GPa, respectively. Arithmetic mean values of T, N and C were 6.73 GPa, 6.13 GPa, and 4.22 GPa for the first half tube wall (near YL) while 24.44 GPa, 20.81 GPa and 15.90 GPa for the latter half (near GL). There is a larger difference on the latter half tube wall than the first half tube wall, while the latter half tube wall bears a higher stress than the first half tube wall during bending.

Our results show that bending has an effect on MOE of the bending section. The MOE value shifts for both tension and compression parts compared with neutral part (Fig. 5c), which is likely due to microstructural changes under bending stress. For example, fiber orientation and degree of crystalline orientation would increase during tension, while decrease during compression^34,42^, which is similar to an increase and a decrease in MOE during the tension and compression phase, respectively^43,44^. The springback in bamboo is caused by multiple factors: (1) uneven distribution of stress along cross section (there is a higher stress in the outside half tube wall than the inside)^41^; (2) the higher MOE in the outside tube wall than the inside; and (3) the higher MOE in the first half bamboo tube wall than the latter half. These characteristics might be helpful to reduce springback for the bending bamboo.

### Correlation between position, density, MFA & MOE

**Figure 6:**
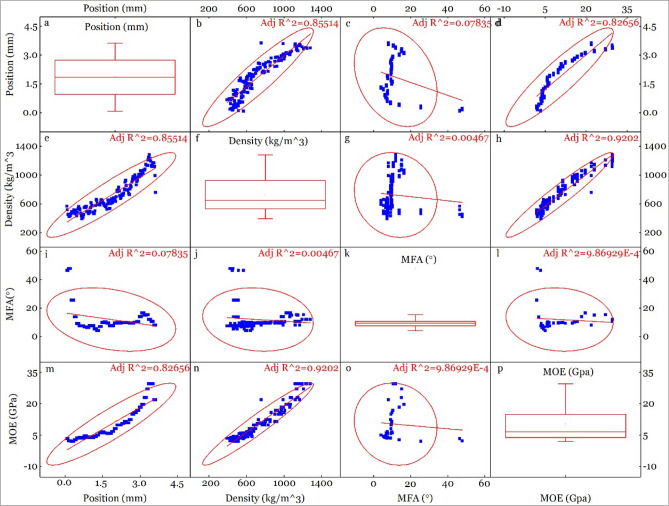
Correlation between position, density, MFA and MOE of compression part.

**Figure 7:**
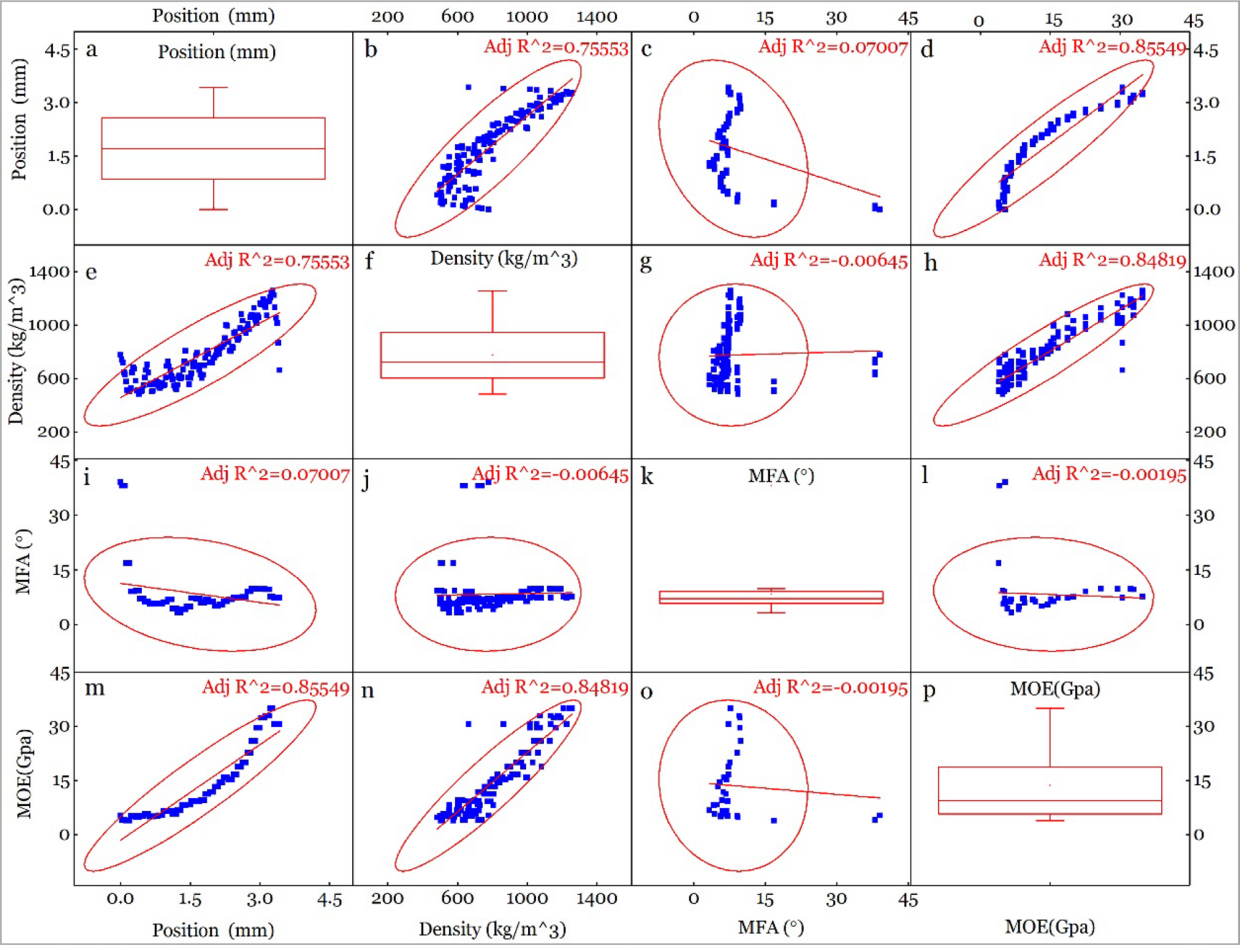
Correlation between position, density, MFA and MOE of neutral part.

**Figure 8:**
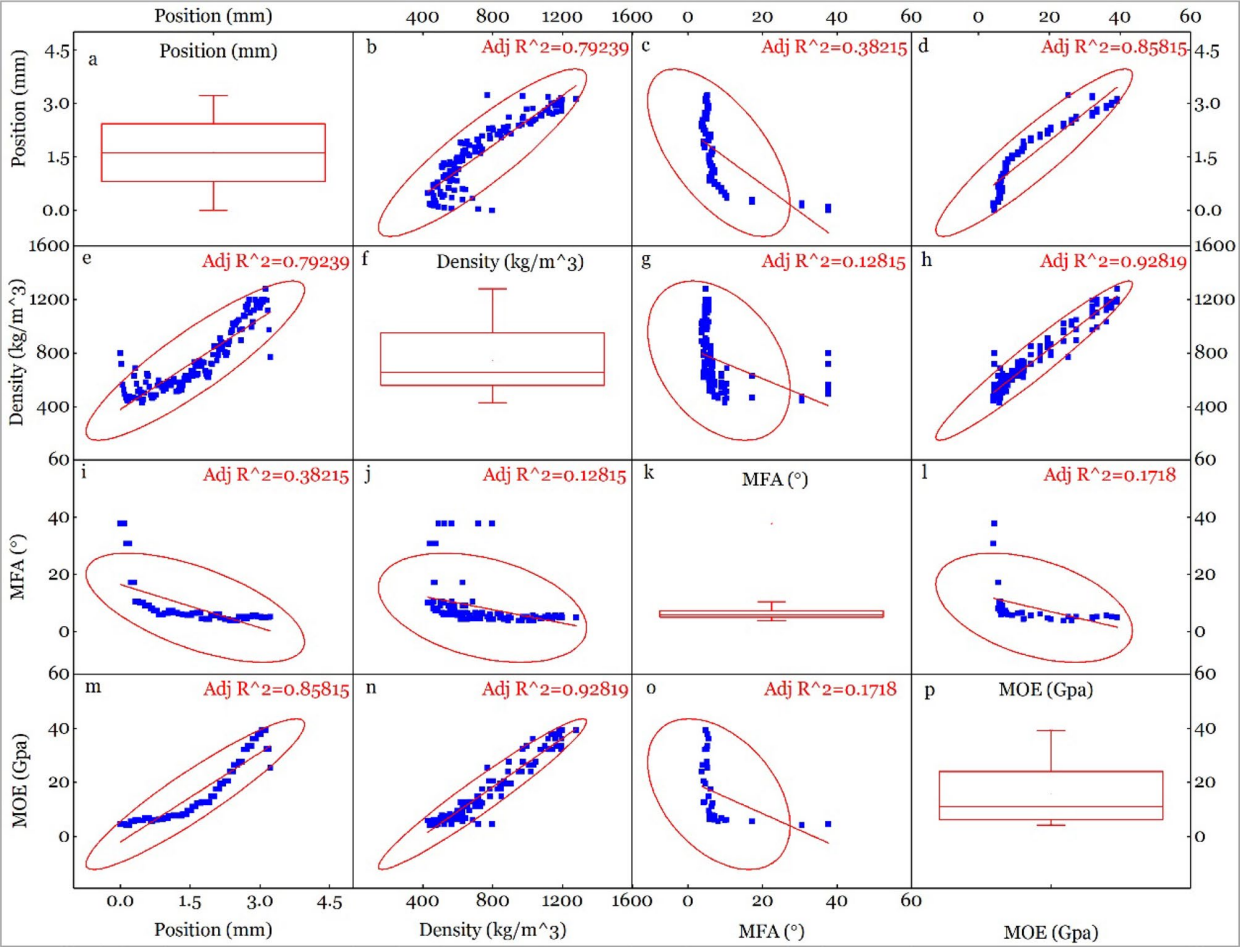
Correlation between position, density, MFA and MOE of tension part.

Correlation between position, density, MFA & MOE of compression (Fig. 6), neutral (Fig. 7) and tension (Fig. 8) parts is figured out. It shows a high correlation between position and density (R^2^ = 0.85514 for C, 0.75553 for N and 0.79239 for T), position and MOE (R^2^ = 0.82656 for C, 0.85549 for N and 0.85815 for T), MOE and density (R^2^ = 0.9202 for C, 0.84819 for N and 0.92819 for T). The high correlation between position and density, position and MOE is caused by the radial distribution of density (Fig. 5a). Density is an important determinant to the strength of bamboo or wood^18,45^, which made it a high correlation with MOE.

MOE is one of the most important mechanical properties for bending applications, which is influenced by density^46^, MFA^47,48^. MFA and density can have different effect on MOE, and is a function of species type. For example, MFA and density jointly account for 96% of the longitudinal MOE variation in *Eucalyptus delegatensis*^49^, while MFA and density, separately account for 87% and 81% of the variation, respectively^50^. Analysis of the relationship between MFA and density have shown negative correlation (− 0.59) in *Pinus taeda L.*^51^. Likewise, MFA and MOE showed larger variation along radial direction (400 kg/m^3^ ~ 1400 kg/m^3^ for density, 5° ~ 48° for MFA and 2 GPa ~ 40 GPa for MOE (Figs. 6, 7 and 8) than wood, indicating that correlations between density, MFA and MOE for the C, N and T part in bending bamboo, might differ with wood.

**Figure 9:**
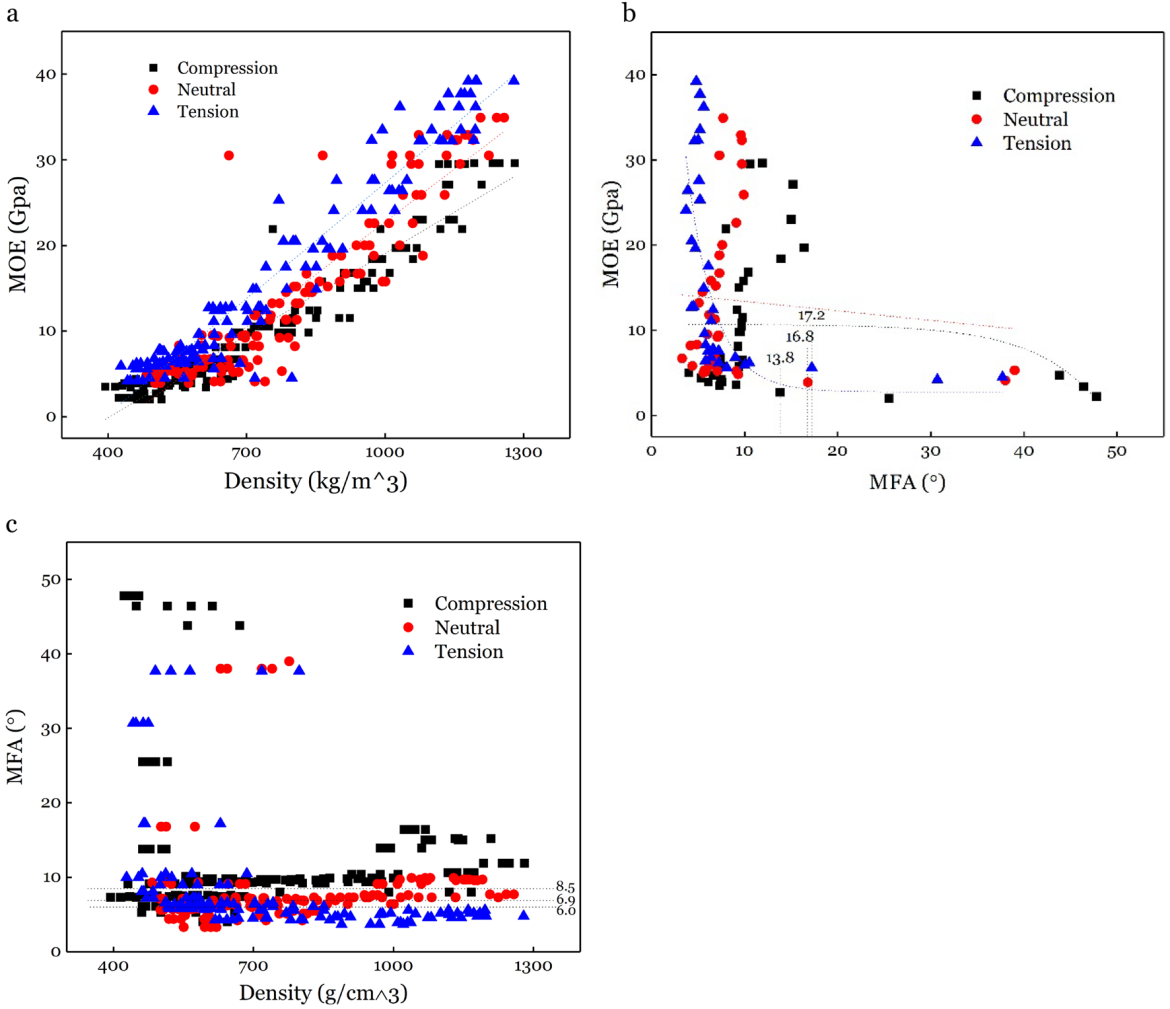
Correlation between (**a**) MOE and density, (**b**) MOE and MFA, and (**c**) MFA and density, of different bamboo cross-section parts.

We found a good line correlation between MOE and density as shown in Figs. 6n, 7n, 8n and 9a, With proportion of variance of R^2^ = 0.9202, 0.84819 and 0.92819 for C, N and N part respectively(Figs. 6, 7, 8, 9). This proportion of variance is higher than R^2^ = 0.54 in moso bamboo, where density and MOE were estimated using drilling resistance technique and static bending test; respectively^52^. Likewise, it show R^2^ = 0.47 in white oak, where density and MOE were analyzed using SilviScan, and R^2^ = 0.70 in *E. delegatensis*, where density and MOE were estimated using SilviScan and vibration testing, respectively^49^. Slope of MOE to density is0.03202, 0.04097 and 0.04501 for compression, neutral and tension part; respectively, which indicates that MOE is more sensitive to density during tension than compression.

While we found a strong linear correlation between MOE and density, there was a negative correlation between MOE and MFA as shown in Fig. 9b. Our results show the R^2^ = 0.42835 for tension part when the expdec1 function was used. However, the correlation was low to meaningless in neutral (R^2^ = − 0.00937) and compression (R^2^ = 0.04631) samples, which indicate that MOE is more sensitive to density in tension part compared to neutral and compression part.

We also found an “impact diminishing” point, where MOE does not reduce further beyond the certain point of MFA. The “impact diminishing” point appears to be 16° in three kinds of eucalyptus species (plantation-grown *E. globulus*, *E. nitens* and *E. regnans*)^50^. In this study, the “impact diminishing” point of the bending bamboo varied as position of the section changed. The “impact diminishing” points were 17.2°, 16.8° and 13.8° for tension, neutral and compression part separately.

A previous study has shown negative correlation between MFA and density in *ineucalyptus globulus* (r = − 0.66) and *Pinus taeda L.* (r = − 0.59)^53^. In this study, there was no obvious linear correlation between MFA and density as shown in Figs. 6j, 7j, 8j and 9c. With some specific high MFA value points when density is below 800 kg/m^3^, MFA approaches to be constant when density changed. After removing the “impact diminishing” and above points, the MFA algebra average value were 6.0°, 6.9° and 8.5° for tension, neutral and compression part, respectively. This shows that MFA declined under tension and rises under compression, which is consistent with MFA changing tendency in tension and compression compared to normal wood^54,55^.

## Conclusion

In this study, we analyzed the density distribution, MFA and MOE to evaluate the bending stability of bamboo. We found that density mainly increased from YL to GL. Our results also showed a decline in mean density for compression (720.38 kg/m^3^) and tension (742.75 kg/m^3^) part compared with neutral part (775.97 kg/m^3^). Analysis of MFA showed variation from 4° to 48° along radial direction, with the highest value (38° ~ 48°) in about 0.2 mm ~ 0.5 mm near yellow bamboo. The MFA declined to 4° ~ 10° in about 0.5 mm to 1.8 mm. Additionally, we found an increase in MFA under the tension sample while MFA decreased under the compression sample (11.0°, 7.5° and 5.0° for C, N and T part separately). MOE also increased from YL to GL, with the order of T > N > C. The differences in MOE value became increasingly larger from 6.73 GPa, 6.13 GPa, and 4.22 GPa in the inner half wall to 24.44 GPa, 20.81 GPa and 15.90 GPa in the outer half for tension, neutral and compression sample, respectively.

Analysis of the relationship between MOE and density showed a strong positive correlation with R^2^ = 0.9202, 0.84819 and 0.92819 for tension, neutral and compression part separately. We also found an inverse relationship between MFA and MOE with “impact diminishing” point of 17.2°, 16.8° and 13.8° for tension, neutral and compression part separately, after which there was no reduction in the MOE. There was no obvious linear relationship between MFA and density. However, after removing some specific high MFA value points when density is below 800 kg/m^3^, MFA values were static with the mean of 6.0°, 6.9°, and 8.5° for tension, neutral and compression part, respectively.
